# Endovascular Treatment of Femoro-Popliteal Disease with the Supera Stent: A Single Center Experience

**DOI:** 10.3390/jcm14051704

**Published:** 2025-03-03

**Authors:** Borivoje Lukic, Marko Miletic, Stefan Milosevic, Marko Dragas, Jovica Saponjski, Igor Koncar, Petar Zlatanovic, Filip Lukic, Aleksandar Mirkovic, Dimitrije Lazic, Ksenija Markovic, Natasa Milic, Vladimir Cvetic

**Affiliations:** 1 Cardiovascular Radiology Department, University Clinical Center of Serbia, Dr. Koste Todorovica 8, 11000 Belgrade, Serbia; 2 Center for Radiology, University Clinical Center of Serbia, Pasterova 2, 11000 Belgrade, Serbia; 3 Faculty of Medicine, University of Belgrade, Dr. Subotica 8, 11000 Belgrade, Serbia; 4 Department of Clinic for Vascular and Endovascular Surgery, University Clinical Center of Serbia, Dr. Koste Todorovica 8, 11000 Belgrade, Serbia; 5 Radiology Department, University Hospital Medical Center Bezanijska Kosa, 11000 Belgrade, Serbia; 6 Institute for Medical Statistics and Informatics, Faculty of Medicine, University of Belgrade, 11000 Belgrade, Serbia; 7 Division of Nephrology and Hypertension, Mayo Clinic, Rochester, MN 55905, USA

**Keywords:** peripheral artery disease, femoropopliteal lesions, biomimetic stents, SUPERA stent, endovascular intervention, patency rates, major adverse limb events

## Abstract

**Background/Objectives**: Peripheral artery disease (PAD) is a significant global health challenge, affecting millions worldwide. Among its various manifestations, femoropopliteal atherosclerotic disease presents a unique challenge due to the biomechanical stresses on the superficial femoral artery (SFA) and popliteal artery (PA). Despite advancements in endovascular interventions, restenosis and stent fractures remain critical issues, particularly in complex and long lesions. Biomimetic stents, such as the SUPERA interwoven nitinol stent, have been developed to address these challenges by closely replicating the natural mechanical properties of the femoropopliteal arteries. This study evaluates the clinical and procedural outcomes of biomimetic stent implantation in patients with femoropopliteal atherosclerotic disease, focusing on patency rates, procedural success, and major adverse limb events (MALE). **Methods:** A cohort study was conducted at the University Clinical Center of Serbia, including 294 patients with femoropopliteal stenosis or occlusion treated with the SUPERA stent from January 2017 to December 2024. Patients were stratified by lesion complexity using the GLASS classification and procedural success, patency rates, and MALE incidence were assessed. Kaplan–Meier survival analysis was used to evaluate long-term outcomes, and Cox regression analysis identified predictors of MALE. **Results:** Primary patency rates at 1, 6, 12, and 24 months were 95.6%, 90.1%, 84.2%, and 77.7%, respectively. Primary-assisted patency and secondary patency rates remained high over time. Patients with GLASS IV lesions exhibited significantly lower patency rates and higher MALE incidence compared to GLASS I-III patients (*p* = 0.002). Occlusion length (≥16 cm) and lesion complexity (GLASS IV) were independent predictors of MALE (*p* = 0.015). The stent demonstrated high procedural success and durability, with minimal complications. **Conclusions:** Biomimetic SUPERA stents provide high patency rates and favorable clinical outcomes in complex femoropopliteal lesions. However, lesion complexity and occlusion length significantly impact long-term success. The findings highlight the importance of careful patient selection and lesion assessment for optimizing endovascular treatment strategies in PAD management.

## 1. Introduction

Peripheral artery disease (PAD) is a significant global health challenge, affecting an estimated 200 million individuals worldwide and serving as a major cause of morbidity and reduced quality of life [1]. PAD is a multifactorial condition associated with high morbidity and mortality, with an increasing incidence in Western countries due to the increasing incidence of chronic pathological conditions, such as type II diabetes mellitus [2]. Among the various manifestations of PAD, femoropopliteal atherosclerotic disease represents one of the most challenging regions to perform surgical or interventional radiological procedures due to the constant presence of strong biodynamic forces, a particularly complex subset due to the biomechanical demands placed on the superficial femoral artery (SFA) and proximal popliteal artery [3]. These vessels experience substantial mechanical forces, including torsion, compression, and flexion, which contribute to the challenges of maintaining long-term vessel patency following endovascular interventions [3]. With the advancement of endovascular treatments for femoropopliteal disease, more sophisticated options, such as new-generation self-expanding nitinol stents, drug-eluting balloons, and drug-eluting stents, have broadened treatment options beyond previous PAD therapies, including balloon angioplasty and first-generation bare-metal stents. While these methods have improved outcomes, restenosis and stent fractures remain prevalent issues, especially in long and complex lesions [4]. There has been a significant increase in the use of endovascular interventions in the treatment of PAD, with femoropopliteal interventions accounting for more than 55% of cases [5]. Although many types of stents have been tested in the SFA and popliteal artery (PA), the outcome of interventions with some types of stents has not been optimal, probably due to inappropriate characteristics that do not match the anatomical and physiological characteristics of this region. First, laser-cut stents have limitations due to the potential for stent rupture and/or buckling, as well as the induction of a significant inflammatory response in the vessel wall. Nevertheless, continued material evolution has led to the development of biomimetic stents that reduce arterial wall stress and strain while providing greater radial strength and fracture resistance [4,6,7]. This has driven the development of biomimetic stents, which aim to address these challenges by closely replicating the natural mechanical behavior of the femoropopliteal arteries [8]. Biomimetic stents, such as the Supera interwoven nitinol stent, feature helical designs that enhance flexibility, radial strength, and fracture resistance, offering a promising alternative to traditional stents [9]. The Supera peripheral stent (Abbott Vascular, Santa Rosa, CA, USA) is a device consisting of six pairs of closed-loop, coiled nitinol wires specifically designed to be durable, flexible, and resistant to damage and breakage. These stent characteristics contribute to the improvement of the treatment of atherosclerotic lesions of the femoropopliteal segment [2]. Despite their promising benefits, the success of biomimetic stents depends on proper implantation techniques and lesion-specific considerations. Studies have shown that suboptimal deployment or implantation defects can negatively impact outcomes, underscoring the importance of operator expertise [7]. Several clinical trials and registry studies have highlighted the advantages of biomimetic stents in improving primary patency rates, reducing the risk of restenosis, and achieving better long-term outcomes [4,10]. However, the success of these interventions depends not only on stent design but also on patient-specific factors, lesion characteristics, and procedural techniques. This study evaluates the success of endovascular treatment of femoropopliteal atherosclerotic disease with biomimetic stents, analyzing clinical and procedural outcomes.

## 2. Materials and Methods

### 2.1. Study Population

This cohort study included patients with stenosis or occlusion of the femoropopliteal segment who have been treated endovascularly with the implantation of a Supera stent between January 2016 and December 2023 at the Department of Cardiovascular Radiology of the University Clinical Center of Serbia in Belgrade. The inclusion criteria were patients with stenosis or occlusive lesions of the femoropopliteal segment of I-IV grade according to the GLASS classification, who have been treated endovascularly with the implantation of one or more biomimetic stents and another type of graft material needed to cover the affected segment of the artery. The exclusion criteria for the study were patients with stenotic or occlusive lesions of the femoropopliteal segment who have been treated endovascularly in combination with surgical treatment (hybrid procedures), patients treated surgically, patients where it has been assessed that necrosis and infection have reached an irreversible stage, and patients with acute thromboembolic limb ischemia. The study was approved by Ethics Committee of Faculty of Medicine University of Belgrade (n:27/I-3).

Pre-procedural assessment of femoropopliteal segment patency was evaluated radiographically using multidetector computed tomography angiography (MDCTA). Based on MDCTA, patients were grouped according to the GLASS classification.

### 2.2. Procedure Details

Patients were selected for endovascular procedures based on clinical evaluation and computed tomography angiography (CTA). All interventions were performed in accordance with institutional protocols, by two experienced interventional radiologists, each with over 15 years of experience in treating PAD and pre-training in optimal stent deployment. The procedures were conducted under local anesthesia, while patients remained conscious. Vascular access was established using a 6F sheath via either contralateral or ipsilateral approach. Following access, anticoagulation with 50 IU/kg of heparin was administered. An initial angiographic assessment with contrast medium was performed to delineate vascular anatomy and lesion characteristics.

In cases where intraluminal crossing was feasible, this approach was prioritized. If subintimal passage was necessary, distal reentry was assessed using contrast injection. Once the lesion was successfully crossed with a guidewire, balloon dilatation was conducted in all patients to prepare the vessel for stent implantation. The balloon size was selected based on preprocedural CTA measurements of the arterial diameter. The decision to implant a Supera stent was individualized by the treating vascular specialist, with indications including subintimal crossing, dissection, recoil, residual stenosis greater than 30%, or primary stenting in cases of severe calcification and complex lesions with high risk of complications. Stent diameters were chosen to match the vessel diameter in a 1:1 ratio, with lesions covered along with at least 5 mm of adjacent healthy vessel. In cases requiring multiple stents, an overlap of at least 10 mm was ensured. Supera was provided on a 6F or 7F delivery system and the shaft lengths varied from 80 cm to 120 cm. The system could be delivered over 0.014 or 0.018 guide wires. Stent diameters from 4.5 mm to 6.5 mm were used, and length from 60 mm to 200 mm. Access site closure was achieved either through manual compression or with closure devices (ANGIO-SEAL^®^ VIP Vascular Closure Device, Terumo, (Terumo Medical Canada Inc., Vaughan, ON, Canada) or Perclose ProGlide^®^ Suture-Mediated Closure System, Abbott (Abbott Vascular, Redwood City, CA, USA)). Post-procedure, all patients were prescribed dual antiplatelet therapy (clopidogrel and aspirin) for a minimum of 90 days. Non-CLTI patients were primarily considered for the endovascular approach. In CLTI, there was no preferred option for the treatment of complex femoropopliteal lesions between endovascular and vein infrainguinal bypass surgery. The patients were evaluated by interdisciplinary vascular experts and the most suitable option for them was selected. All procedures were performed with optimal stent deployment. No stent fractures were recorded.

### 2.3. Outcomes

Primary outcomes were patency rates, including primary patency, assisted primary patency, and secondary patency rates. In addition, freedom from bypass surgery and freedom from MALE were also calculated, as well as overall survival. Patency rates were assessed according to arteries of stents implants (SFA, PA and both).

### 2.4. Definitions

Primary patency rate was defined as a lack of clinical deterioration or significant restenosis evaluated in ultrasound follow-up. In ultrasound follow-up, the definition of significant restenosis was peak systolic velocity > 250 cm/s or proximal to distal peak systolic velocity ratio > 2.5, or a monophasic waveform in the artery distal to the stent. Assisted primary patency was defined as the patency rate of an intervention that required reintervention but did not progress to thrombosis. Secondary patency referred to the patency rate of a procedure that failed to the point of thrombosis but was successfully restored through reintervention. Technical success was defined as the restoration of vessel patency with residual stenosis of less than 30%. Procedural success was characterized by achieving technical success without any complications during the procedure. Major amputation was classified as the amputation of the lower limb at or above the level of the ankle.

Stenosis was defined as luminal narrowing with a diameter stenosis > 70% on angiography. CTO was defined as complete occlusion of the arterial segments for more than 3 months. Intermittent claudication was characterized as pain localized to the calf during walking, absent during sitting or standing, and which persists while walking, necessitating cessation or slowing of activity, and resolving within 10 min of rest. Chronic limb-threatening ischemia (CLTI) was defined by the presence of rest pain, ulcer, or gangrene associated with confirmed arterial occlusive disease. Patients were categorized into the renal insufficiency group if their glomerular filtration rate (GFR) was less than 60. Length of the lesions were classified as ≤5, 6–10, 11–15, 16–20, and >20. Calcification degree was categorized as either absence or presence of calcification. Ankle-brachial index (ABI) was performed.

### 2.5. Clinical Follow-Up

Immediate post-procedural patency was evaluated radiographically using digital subtraction angiography (DSA). Peri-procedural complications during 24 h after implantation were also analyzed. Clinical follow-up consisted of periodic visits before hospital discharge, at 1 month, 6, 12, and 24 months after the procedure. Follow-up assessments included patient physical examination and measuring the ankle-brachial index (ABI), palpation of popliteal and pedal pulses, Doppler ultrasound, and if necessary, MDCTA or DSA.

### 2.6. Statistical Analysis

Numerical variables are presented as mean ± standard deviation or as median (range). Categorical variables are given as count (percentage). Outcomes such as primary patency rate, primary assisted patency rate and secondary patency rate, bypass freedom, freedom from MALE, and survival were calculated using Kaplan–Meier survival analyses within a 24 month period. In the case of an event, the time of the event was recorded. For censored observations without an event, the time of the last follow-up was used. Standard Errors (SE) and 95% Confidence Intervals (95% CI) were calculated. Differences in Kaplan–Meier curves were evaluated between patency rates, bypass freedom, MALE, and death during a 24 month follow up. Differences between groups in patency rates were evaluated with the log-rank test. Univariate and multivariate Cox regression analysis was used for identifying predictors of MALE in patients with occlusive lesions of the femoropopliteal segment treated with Supera stent. Statistical significance was conducted for the two-sided *p*-value < 0.05. Statistical analysis was conducted using IBM SPSS Statistics 25 software (IBM SPSS Inc., Chicago, IL, USA).

## 3. Results

The study population consisted of 294 patients with a mean age of 68.54 ± 9.48 years, with 66.0% being male. The prevalence of comorbidities included diabetes mellitus (61.9%), hypertension (85.4%), and hypercholesterolemia (48%). Smoking history revealed that 46.3% were current smokers, while 21.4% were overweight (Table 1).

According to the WIFI (Wound, Ischemia, and Foot Infection) risk classification, 35.0% of patients had a very low or low amputation risk, 33.3% had a moderate risk, and 31.6% had a high risk, depending on the condition of both legs. According to WIFI revascularization needs, 48.6% were high risk, 32.7% were intermediate risk, and 18.7% were extremely low or low risk. Gangrene affected 28.9% of patients, ulceration affected 33.7%, and rest discomfort affected 67.7% of patients. The ankle-brachial index (ABI) values revealed that 20.4% of patients had an ABI of <0.25, and 42.2% had values between 0.25 and 0.35. Additionally, distal runoff was preserved in 89.5% of cases, while 10.5% showed no distal runoff. Crural patency was absent in 10.5% of cases, while patency of one, two, or three crural arteries was represented in 38.8%, 38.8%, and 11.9% of cases, respectively. A total of 66.3% of cases had calcified lesions and 86.1% had chronic total occlusion (CTO) lesions (Table 2).

The most common occlusion length was 6–10 cm (30.6%), and the majority of lesions were located in both the superficial femoral artery and popliteal artery (42.2%). The diseased segment length was >20 cm in 27.2% of cases. GLASS classification identified 48.6% of cases as GLASS IV. The Supera stent used in this study has the most frequent stent diameter 4–5.5 mm (71.8%), and stent lengths ranged from 10–18 cm in most cases (69.3%) (Table 3).

### Clinical Outcomes by Kaplan–Meier Estimates and Predictors of MALE

**Figure 1:**
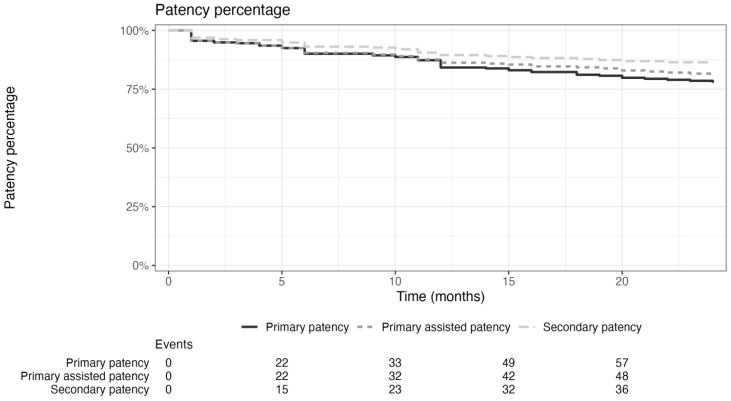
Kaplan–Meier curve for primary patency, primary-assisted patency, and secondary patency of Supera stent implantation at 24 months follow-up.

Primary patency rates were 95.6%, 90.1%, 84.2%, and 77.7% at 1, 6, 12, and 24 months, respectively. Primary assisted patency rates were 95.6%, 90.4%, 86.3%, and 81.6% at 1, 6, 12, and 24 months, respectively. Secondary patency rates showed a similar decline, with 96.9% at 1 month and 86.5% at 24 months (Figure 1). Bypass-free survival remained high, ranging from 99.3% at 1 month to 98.2% at 24 months. Amputation-free survival declined modestly from 98.6% at 1 month to 92.6% at 24 months. Major adverse limb events (MALE) occurred in 4.4% of cases at 12 months, increasing to 22.3% at 24 months. Death rates were very low at all time points, ranging from 1% at 1 month to 1.8% at 24 months, demonstrating the low procedural mortality risk (Table 4).

Most patients included in the study had CTLI (87.8%, n = 258), while 12.2% had intermittent claudication (n = 36). Table 5 presents Kaplan–Meier estimates for primary patency, primary assisted patency, secondary patency, bypass freedom, MALE, and death rate of Supera stent implantation at 24 months follow-up according to presence of CTLI and intermittent claudication.

GLASS IV lesions presented significantly lower patency rates and freedom from MALE at all time points in contrast to GLASS I–III lesions (Table 6), while freedom from bypass and amputation were not significantly different between groups (Table 5). At 24 months, primary patency was 85.3% for GLASS I–III but significantly lower (69.7%) for GLASS IV. Similar primary-assisted patency GLASS IV outcomes compared to GLASS I–III, with rates at 73.7% vs. 89.0% at 24 months. Secondary patency shows slightly better performance compared to primary assisted patency, particularly for GLASS IV lesions (80.5% at 24 months), where MALE also shows a sharp rise to 22.3% at 24 months, highlighting the growing burden of adverse events for GLASS IV lesions.

**Figure 2:**
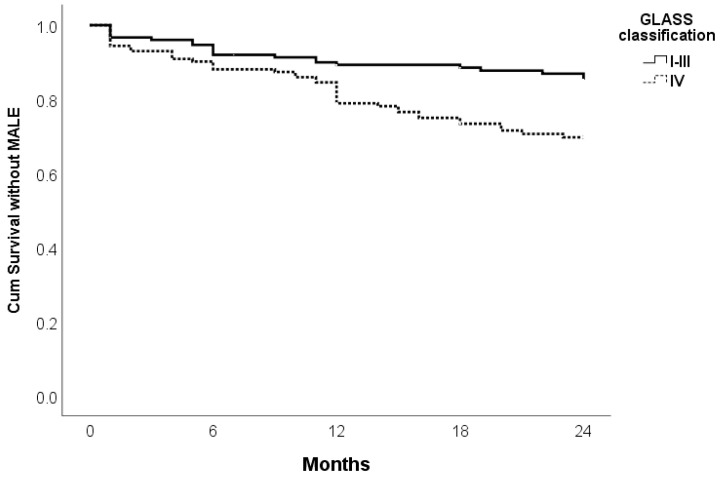
Kaplan Meier curve for survival without MALE of Supera stent implantation at 24 months follow-up according to GLASS classification.

Patients classified under GLASS I–III demonstrate significantly better cumulative survival without MALE compared to those in GLASS IV (*p* = 0.002). Follow-up over 24 months indicates that more advanced stages of the disease (GLASS IV) are associated with worse outcomes (Figure 2).

**Figure 3:**
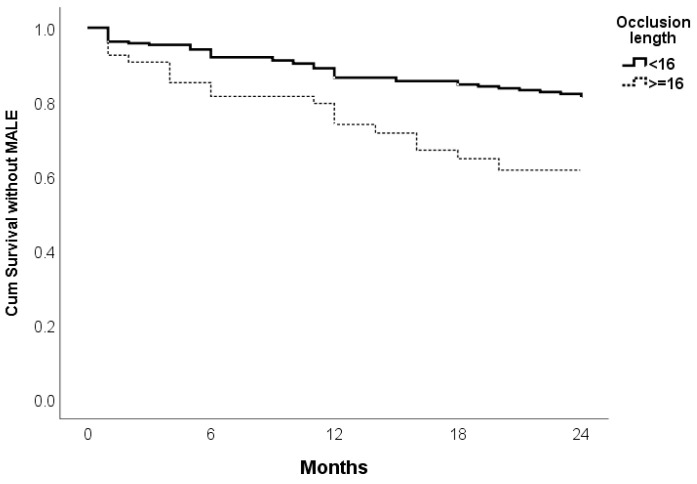
Kaplan–Meier curve for survival without MALE of Supera stent implantation on 24 months follow-up according to occlusion lengths.

There is a significant difference in survival without MALE based on the occlusion length. Patients with occlusion length < 16 cm have superior outcomes compared to those with occlusion length ≥ 16 cm (*p* = 0.002). The curve suggests that longer occlusions are correlated with a greater risk of MALE, leading to a noticeable difference in outcomes between the two groups at the 24 month mark (Figure 3).

**Figure 4:**
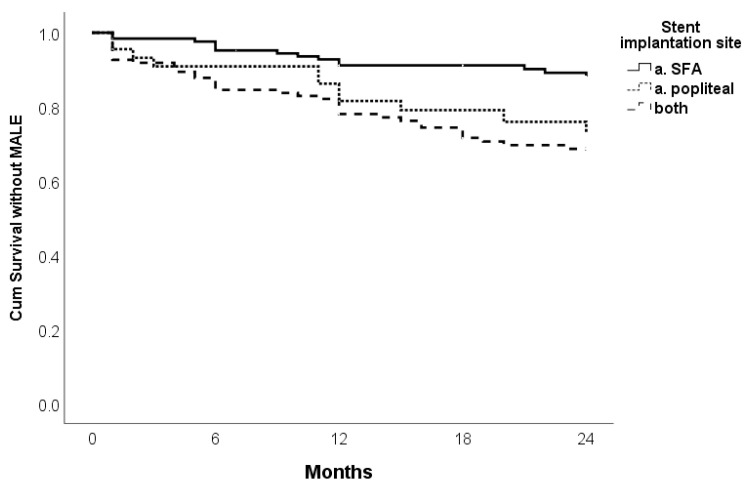
Kaplan–Meier curve for survival without MALE of Supera stent implantation on 24 months follow-up according to stent site implantation.

There was a difference in survival without MALE according to stent site implantation (*p* = 0.001). SFA (Superficial Femoral Artery) shows better cumulative survival without MALE compared to popliteal artery or combined cases (Figure 4). Univariate Cox regression analysis identified WIFI risk for revascularization (HR 1.439, 95% CI 1.010–2.049, *p* = 0.044), occlusion length (HR 2.309, 95% CI 1.343–3.970, *p* = 0.002), GLASS classification (HR 2.223, 95% CI 1.313–3.763, *p* = 0.003) and stent site (HR 2.437, 95% CI 1.106–5.370, *p* = 0.027 and HR 3.000, 95% CI 1.106–5.370, *p* = 0.001 for a. poplitea and both arteries, respectively) as independent predictors of MALE. The multivariate analysis identified occlusion length (HR 1.933, 95% CI 1.108–3.374, *p* = 0.020) and GLASS classification (HR 1.963, 95% CI 1.143–3.373, *p* = 0.015) as the most significant predictors of MALE (Table 6).

Univariate Cox regression analysis identified WIFI risk for revascularization (HR 1.439, 95% CI 1.010–2.049, *p* = 0.044), occlusion length (HR 2.309, 95% CI 1.343–3.970, *p* = 0.002), GLASS classification (HR 2.223, 95% CI 1.313–3.763, *p* = 0.003), and stent site (HR 2.437, 95% CI 1.106–5.370, *p* = 0.027 and HR 3.000, 95% CI 1.106–5.370, *p* = 0.001 for a. poplitea and both arteries, respectively) as independent predictors of MALE. The multivariate analysis identified occlusion length (HR 1.933, 95% CI 1.108–3.374, *p* = 0.020) and GLASS classification (HR 1.963, 95% CI 1.143–3.373, *p* = 0.015) as most significant predictors of MALE (Table 7).

## 4. Discussion

The primary objective of this study was to evaluate the success of maintaining flow patency and the effectiveness of the Supera stent in patients with stenotic or occlusive femoropopliteal lesions. The assessment focused on its performance peri-procedurally (up to 1 month post-procedure) and at follow-up intervals of 6, 12, and 24 months. The Supera stent is specifically designed to replicate the flexibility and dynamic motion of the arterial wall. Its unique interwoven structure provides superior radial strength and fracture resistance, while its high crush resistance ensures durability against external mechanical forces. These biomechanical features offer a distinct advantage, particularly in addressing complex femoropopliteal lesions, which represent one of the most significant challenges in endovascular treatment.

Our study demonstrated primary patency rates of 84.2% at 12 months and 77.7% at 24 months, while assisted primary patency rates were 86.3% at 12 months and 81.6% at 24 months. Secondary patency rates were 89.5% at 12 months and 86.5% at 24 months. These results are consistent with findings from the Leipzig Supera 500 registry, which reported primary patency rates of 83.3% at 12 months and 72.8% at 24 months, along with secondary patency rates of 98.1% at 12 months and 92.0% at 24 months in cases of complex femoropopliteal lesions [8]. Similarly, an Italian multicentric study reported primary patency rates of 83.1%, 74.3%, and 69.5% at 12, 24, and 36 months, respectively, with assisted primary patency rates of 89.9%, 76.8%, and 73.4% over the same periods [11].

These findings underscore the Supera stent’s ability to maintain high patency rates over time, even in anatomically challenging regions such as the femoropopliteal artery. Notably, these studies also highlighted the stent’s capacity to preserve its structural integrity over time, even in high-risk patients with complex lesions.

Schillinger et al. observed that nitinol stents, including the Supera stent, demonstrated enhanced durability in high-compression zones [12]. Additionally, the Zilver PTX randomized trial reported superior outcomes with nitinol stents compared to percutaneous transluminal angioplasty [13]. Beyond its durability, the Supera stent has shown promise in reducing complications such as restenosis and the need for reintervention. Data from multiple registries and randomized controlled trials suggest that the stent’s unique design minimizes endothelial damage and promotes favorable hemodynamic flow, thereby reducing the risk of neointimal hyperplasia [4,7].

The stent’s efficacy in treating infrainguinal PAD has been attributed to its superior radial strength and flexibility, which are particularly advantageous for addressing complex femoropopliteal lesions [14]. Comparative studies on stenting strategies have demonstrated that stents like Supera achieve effective outcomes by significantly lowering restenosis and reintervention rates, especially in long lesions [15].

Previous studies have demonstrated that Supera stents achieve favorable patency rates in managing complex and extensive lesions within the femoropopliteal segment. While most existing research focuses on lesions shorter than 150 mm, only a limited number of studies, including ours, evaluate outcomes in longer lesions [16,17,18,19]. Moreover, the stent’s performance has been assessed in challenging clinical scenarios, such as total occlusions, long lesions exceeding 15 cm, and calcified plaques, where conventional therapies often prove inadequate.

In our study, longer occlusion lengths (≥16 cm) and higher-grade GLASS category (GLASS IV) was associated with lower primary patency rates. Similarly, the STELLA-SUPERA Trial and other studies have reported a higher prevalence of CTOs, aligning with our findings, where 86.1% of the lesions were CTOs [20,21,22]. This observation is consistent with Maleckis et al., who emphasized that lesion length and complexity significantly influence stent performance, underscoring the importance of tailoring treatment strategies to the characteristics of individual lesions [23].

Among nitinol stents, the Supera stent has demonstrated the highest torsional stiffness and radial compressive response, while maintaining relatively low axial stiffness. This balance enhances flexibility and reduces adverse interactions with the arterial wall, making the Supera stent particularly well-suited to withstand the flexion forces characteristic of the femoropopliteal segment. The advancement of endovascular device technology and increasing operator expertise have expanded the indications for minimally invasive procedures, enabling the treatment of more complex lesions [24].

The recent Global Vascular Guidelines for the Management of Chronic Limb-Threatening Ischemia introduced a new anatomical classification system for infrainguinal disease known as the “Global Limb-Anatomic Staging System” (GLASS), replacing the earlier TransAtlantic Inter-Society Consensus II (TASC II) classification. GLASS categorizes femoropopliteal disease into four grades, with higher grades indicating more complex disease, while also incorporating an assessment of the infrapopliteal segment. This updated classification of anatomical lesion patterns (GLASS) is designed to complement patient risk evaluation, which takes into account comorbidities and the clinical stage of limb disease, as determined by the WIFI classification (Wound, Ischemia, and Foot Infection) [25,26].

In our study, patients with GLASS I–III lesions demonstrated a significantly better cumulative survival rate without major adverse limb events (MALE) compared to those with GLASS IV lesions. Primary patency declined more sharply in the GLASS IV group over a 24 month period, indicating that more complex disease stages are associated with poorer outcomes. Patency rates were also significantly lower in lesions extending from the SFA to the PA, whereas outcomes were more favorable in lesions sparing the PA. The increased stress and higher flexion-extension, compression, and rotational forces associated with the PA likely contribute to restenosis and reduced patency rates [10,27,28,29] Although no stent fractures were observed in this study, retrospective data support the hypothesis that mechanical stress plays a significant role in restenosis and the decline in patency.

In addition to patency rates, the incidence of MALE serves as a critical indicator of clinical success in femoropopliteal interventions. In our study, the MALE rate at 24 months was 22.3%, aligning with results from other studies evaluating the Supera stent. Similarly, the SUPERB trial reported a 12 month primary patency rate of 86.3% and a freedom from target lesion revascularization rate of 90.5%, highlighting the favorable performance of the Supera stent [9]. These findings indicate that the Supera stent is associated with acceptable MALE rates, particularly in the treatment of complex femoropopliteal lesions. However, it is important to acknowledge that clinical outcomes can vary depending on lesion complexity, patient comorbidities, and procedural factors.

### Limitations

Despite these promising results, the use of the Supera stent is not without limitations. The technical challenges involved in deploying the stent, such as precise positioning and avoiding oversizing, require specialized training and expertise. While our study provides valuable insights, it is limited by its retrospective design and the absence of a control group (e.g., alternative therapies such as drug-coated balloons and drug-eluting stents). Additionally, long-term outcomes beyond 24 months were not assessed. Therefore, further research is warranted to evaluate the long-term outcomes and cost-effectiveness of the Supera stent in comparison with alternative therapies, including drug-coated balloons and drug-eluting stents. Future randomized studies with extended follow-up periods are needed to validate these findings.

## 5. Conclusions

In conclusion, our study demonstrated patency rates for Supera stent similar to those reported in recent literature. The stent’s performance appears to be influenced by the site, length, and complexity of the lesion, underscoring the importance of careful patient and lesion assessment in clinical decision-making. This study also highlights the value of the GLASS classification in predicting long-term outcomes and emphasizes the need for the development of targeted strategies to manage high-risk (GLASS IV) patients.

## Figures and Tables

**Table 1:** Demographic data and comorbidities of study population.

	n (%)
**Age (med, range)**	68.54 ± 9.48
**Gender**	
**Male**	194 (66.0)
**Female**	100 (34.0)
**Smoking**	
**No**	95 (32.3)
**Former**	63 (21.4)
**Current**	136 (46.3)
**Obesity**	63 (21.4)
**DM**	182 (61.9)
**Insulin treatment**	96 (32.7)
**HTN**	251 (85.4)
**Hypercholesterolemia**	141 (48)
**IHD**	70 (23.8)
**CHF**	20 (6.8)
**Renal insufficiency**	27 (9.2)
**Previous CV**	104 (35.4)

**Table 2:** Lesion characteristics of studied population.

	n (%)
**WiFi amputation risk**	
Very low/low	103 (35.0)
Moderate	98 (33.3)
High	93 (31.6)
**WiFi revascularization**	
Very low/low	55 (18.7)
Moderate	96 (32.7)
High	143 (48.6)
**Rest pain**	199 (67.7)
**Ulcer**	99 (33.7)
**Gangrene**	85 (28.9)
**ABI**	
<0.25	60 (20.4)
0.25–0.35	124 (42.2)
0.4–0.7	76 (25.9)
>1.2 or amputation	34 (11.6)
**ABI other leg**	
<0.25	7 (2.4)
0.25–0.35	37 (12.6)
0.4–0.7	153 (52.0)
0.75–1.2	65 (22.1)
>1.2 or amputation	32 (10.9)
**Distal run off**	263 (89.5)
**Crural not patent**	31 (10.5)
**Crural patent number arteries**	
1	114 (38.8)
2	114 (38.8)
3	35 (11.9)
**Calcified lesion**	195 (66.3)
**CTO lesion**	253 (86.1)

**Table 3:** Diseased segment size, stent size, and GLASS classification.

	n (%)
**Occlusion length (cm)**	
0–5	65 (22.1)
6–10	90 (30.6)
11–15	85 (28.9)
16–20	34 (11.6)
20	20 (6.8)
**Diseased segment**	
a. SFA	123 (41.8)
a. Poplitea	47 (16.0)
Both	124 (42.2)
**Diseased segment length (cm)**	
<10	52 (17.7)
10–20	162 (55.1)
>20	80 (27.2)
**GLASS femoropopliteal classification**	
1	5 (1.7)
2	21 (7.1)
3	125 (42.5)
4	143 (48.6)
**GLASS crural classification**	
0	20 (6.8)
1	60 (20.4)
2	92 (31.3)
3	82 (27.9)
4	40 (13.6)
**Stent implantation site**	
a. SFA	126 (42.9)
a. Poplitea	45 (15.3)
Both	123 (41.8)
**Diameter of stent (mm)**	
4, 4.5/5, 5.5	211 (71.8)
6/6.5	83 (28.2)
**Length of stent (cm)**	
6–8	55 (18.7)
10–12	97 (33.0)
15–18	107 (36.4)
20	35 (11.9)
**Additional stent**	61 (20.7)
Supera	13 (4.4)
Other	41 (13.9)
Both	7 (2.4)
**Inflow vess PTA stent**	16 (5.4)
**Access point complication postop**	8 (2.7)
**Technical success stenting crural**	62 (97.3)
**Technical success stenting femoropopliteal**	294 (100.0)

**Table 4:** Kaplan–Meier estimates presenting overall primary patency, primary assisted patency, secondary patency, bypass freedom, MALE, and death rate of Supera stent implantation at 24 months follow-up.

	1 Month	6 Months	12 Months	24 Months
**Primary patency**	95.6%	90.1%	84.2%	77.7%
	(93.2–97.9%)	(86.7–93.4%)	(80.1–88.3%)	(72.8–82.6%)
**Primary assisted patency**	95.6%	90.4%	86.3%	81.6%
	(93.2–97.9%)	(87.0–93.7%)	(82.4–90.2%)	(77.1–86.1%)
**Secondary patency**	96.9%	93.1%	89.5%	86.5%
	(94.9–98.8%)	(90.1–96.0%)	(85.9–93.0%)	(82.4–90.6%)
**Bypass freedom**	99.3%	98.6%	98.2%	98.2%
	(98.3–100.0%)	(97.2–100.0%)	(96.6–99.8%)	(96.6–99.8%)
**Amputation free**	98.6%	98.0%	95.9%	92.6%
	(97.2–100.0%)	(96.0–100.0%)	(93.5–98.2%)	(89.5–95.7%)
**MALE free**	95.6%	90.1%	87.3%	77.7%
	(93.2–97.9%)	(86.8–93.4%)	(83.6–91.0%)	(72.8–82.6%)
**Survival**	99%	99%	98.3%	98.3%
	(97.8–100.0%)	(97.8–100.0%)	(96.7–99.9%)	(96.7–99.9%)

Numbers in parentheses are 95% confidence intervals.

**Table 5:** Outcomes at 1, 6, 12, and 24 months according to the presence of CLTI or intermittent claudication.

Outcome	1 Month		6 Months		12 Months		24 Months	
	CLTI	IC	CLTI	IC	CLTI	IC	CLTI	IC
**Primary patency**	95.3	97.2	89.1	97.2	83.6	88.9	76.6	85.6
	(92.7–97.8)	(91.9–100.0)	(85.4–92.8)	(91.9–100.0)	(79.1–88.1)	(78.7–99.1)	(71.3–81.9)	(73.8–97.4)
**Primary ass. patency**	95.3	97.2	89.5	97.2	85.9	88.9	80.5	88.9
	(92.7–97.8)	(91.9–100.0)	(85.8–93.2)	(91.9–100.0)	(81.6–90.2)	(78.7–99.1)	(75.4–85.6)	(78.7–99.1)
**Secondary patency**	96.5	100.0	92.1	94.3	88.8	94.3	85.3	94.3
	(94.3–98.6)	/	(88.8–95.4)	(86.6–100.0)	(84.9–92.7)	(86.6–100.0)	(80.8–89.8)	(86.0–100.0)
**By pass freedom**	99.2	100.0	98.4	100.0	98.0	100.0	98.0	100.0
	(98.2–100.0)	/	(96.8–99.9)	/	(96.2–99.8)	/	(96.2–99.8)	/
**Amputation**	98.4	100.0	96.5	100.0	95.3	100.0	92.0	96.7
	(96.8–99.9)	/	(94.3–98.6)	/	(92.7–97.8)	/	(88.5–95.5)	(90.2–100.0)
**MALE**	95.3	97.2	91.8	97.2	83.6	88.9	76.6	85.6
	(92.7–97.8)	(91.9–100.0)	(88.5–95.1)	(91.9–100.0)	(79.1–88.1)	(78.7–99.1)	(71.3–81.9)	(73.8–97.4)
**Death**	98.8	100.0	98.8	100.0	98.1	100.0	98.1	100.0
	(97.4–100.0)	/	(97.4–100.0)	/	(96.3–99.9)	/	(96.3–99.9)	/

Numbers in parentheses are 95% confidence intervals. CLTI, chronic limb threatening ischemia; IC, intermittent claudication.

**Table 6:** Outcomes at 1, 6, 12 and 24 months by GLASS classification.

Outcome	1 Month		6 Months		12 Months		24 Months	
	GLASS 1–3	GLASS 4	GLASS 1–3	GLASS 4	GLASS 1–3	GLASS 4	GLASS 1–3	GLASS 4
**Primary patency**	96.7%	94.4%	92.0%	88.1%	89.3%	78.9%	85.3%	69.7%
	(93.8–99.6%)	(90.7–98.1%)	(87.7–96.3%)	(82.8–93.4%)	(84.4–94.2%)	(72.2–85.6%)	(79.4–91.2%)	(61.8–77.5%)
**Primary ass. patency**	96.7%	94.4%	92.7%	88.1%	90.6%	81.7%	89.0%	73.7%
	(93.8–99.6%)	(90.7–98.1%)	(88.6–96.8%)	(82.8–93.4%)	(85.9–95.3%)	(75.4–88.0%)	(83.9–94.1%)	(66.0–81.3%)
**Secondary patency**	98.7%	95.1%	95.2%	90.8%	93.8%	85.0%	92.1%	80.5%
	(96.9–100.4%)	(91.6–98.6%)	(91.7–98.7%)	(86.1–95.5%)	(89.8–97.7%)	(79.1–90.9%)	(87.6–96.6%)	(73.6–87.3%)
**By pass freedom**	99.3%	95.6%	96.0%	94.3%	95.3%	90.7%	95.3%	90.7%
	(97.9–100.6%)	(92.7–98.5%)	(92.9–99.1%)	(90.6–98.0%)	(92.0–98.6%)	(85.8–95.6%)	(92.0–98.6%)	(85.8–95.6%)
**Amputation**	100.0%	97.2%	98.7%	95.1%	98.0%	93.7%	94.8%	90.4%
	(/)	(94.4–99.9%)	(96.9–100%)	(91.6–98.6%)	(95.6–100%)	(89.8–97.6%)	(91.1–98.5%)	(85.3–95.5%)
**MALE**	96.7%	94.4%	92.0%	88.1%	89.3%	78.9%	85.3%	69.7%
	(93.8–99.6%)	(90.7–98.1%)	(87.7–96.3%)	(82.8–98.4%)	(84.4–94.2%)	(72.2–85.6%)	(79.4–91.2%)	(61.8–77.5%)

Numbers in parentheses are 95% confidence intervals.

**Table 7:** Cox regression univariate and multivariate analysis with MALE as dependent variable.

	Univariate			Multivariate		
	*p*	HR	95% CI	*p*	HR	95% CI
WiFi revascularization	0.044	1.439	1.010–2.049			
Occlusion length	0.002	2.309	1.343–3.970	0.020	1.933	1.108–3.374
GLASS I–III vs. IV	0.003	2.223	1.313–3.763	0.015	1.963	1.143–3.373
Stent site						
SFA, reference	/	/	/			
a. Poplitea	0.027	2.437	1.106–5.370			
a. SFA+ a. Poplitea	0.001	3.000	1.622–5.550			

## Data Availability

The data presented in this study are available on request from the corresponding author due to privacy reasons.
